# Antithrombotic treatment in elderly patients with atrial fibrillation: a practical approach

**DOI:** 10.1186/s12872-015-0137-7

**Published:** 2015-11-04

**Authors:** Carmen Suárez Fernández, Francesc Formiga, Miguel Camafort, Jose María Cepeda Rodrigo, Jesús Díez-Manglano, Antonio Pose Reino, Gregorio Tiberio, Jose María Mostaza, on behalf of Grupo de trabajo de Riesgo vascular de la SEMI

**Affiliations:** Hospital Universitario de La Princesa, Grupo de Riesgo Vascular de la SEMI, Madrid, España; Hospital Universitari de Bellvitge, Grupo de Riesgo Vascular de la SEMI, Hospitalet de Llobregat, Barcelona, España; Atrial Fibrillation Unit (UFA), Internal Medicine Department, Hospital Clinic. University of Barcelona. Research Group in Cardiovascular Risk, Nutrition and Aging. Area. ‘August Pi i Sunyer‘ Biomedical Research Institute (IDIBAPS), Barcelona, Spain; Hospital Vega Baja de Orihuela, Grupo de Riesgo Vascular de la SEMI, Orihuela, Alicante España; Hospital Royo Villanova, Grupo de Riesgo Vascular de la SEMI, Zaragoza, España; Complexo Hospitalario Universitario de Santiago, Grupo de Riesgo Vascular de la SEMI, Santiago de Compostela, España; Hospital Virgen del Camino, Grupo de Riesgo Vascular de la SEMI, Pamplona, España; Hospital Carlos III, Grupo de Riesgo Vascular de la SEMI, Madrid, España; Servicio de Medicina Interna, Hospital Universitario de La Princesa, C/Diego de León 62, 28006 Madrid, Spain

**Keywords:** Anticoagulation, Antithrombotic therapy, Apixaban, Atrial fibrillation, Dabigatran, Direct oral anticoagulants, Edoxaban, Elderly, Rivaroxaban, Warfarin

## Abstract

**Background:**

Atrial fibrillation (AF) in the elderly is a complex condition. It has a direct impact on the underuse of antithrombotic therapy reported in this population.

**Discussion:**

All patients aged ≥75 years with AF have an individual yearly risk of stroke >4 %. However, the risk of hemorrhage is also increased. Moreover, in this population it is common the presence of other comorbidities, cognitive disorders, risk of falls and polymedication. This may lead to an underuse of anticoagulant therapy. Direct oral anticoagulants (DOACs) are at least as effective as conventional therapy, but with lesser risk of intracranial hemorrhage. The simplification of treatment with these drugs may be an advantage in patients with cognitive impairment.

The great majority of elderly patients with AF should receive anticoagulant therapy, unless an unequivocal contraindication. DOACs may be the drugs of choice in many elderly patients with AF.

**Summary:**

In this manuscript, the available evidence about the management of anticoagulation in elderly patients with AF is reviewed. In addition, specific practical recommendations about different controversial issues (i.e. patients with anemia, thrombocytopenia, risk of gastrointestinal bleeding, renal dysfunction, cognitive impairment, risk of falls, polymedication, frailty, etc.) are provided.

## Background

Atrial fibrillation (AF) is a common disease in the elderly. Data from ATRIA (United States) and VAL-FAAP (Europe) studies have shown that 9 % and 17.6 %, respectively, of patients aged 80 years or older have this condition [1, 2].

In AF patients, those individuals aged ≥ 75 years have a worse prognosis compared with subjects between 65 and 74 years, with higher mortality and major adverse cardiac events rates [3]. In fact, in a cohort of individuals aged ≥75 years with AF in Sweden, mortality reached 18.2 % [4].

The risk of stroke is increased four- to five-fold in patients with AF, particularly in elderly. Whereas AF accounts for ≥15 % of all strokes in United States, these numbers increase to 36 % for individuals aged >80 years old. Remarkably, ischemic strokes associated to AF are more likely to be chronically disabled, bedridden, and to require constant nursing care, particularly in elderly [5]. Therefore, prevention of stroke is the most important goal in these patients. In this context, anticoagulation should be considered for most patients. However, age is not only an independent predictor of stroke in AF patients, but also of bleeding risk [6].

AF is a complex condition in elderly subjects due to their high number of comorbidities, including cardiovascular and kidney diseases, cognitive disorders, falls and polypharmacy [7]. As a result, the management of these patients is challenging in most cases. Although the use of anticoagulants has increased in the last years, they are still underused in elderly population [8–11].

The aim of this update was to review the available evidence about the management of anticoagulation in elderly patients with AF and provide some practical recommendations about different controversial issues.

## Evidence and recommendations

### The answers to each question are presented here

#### Antithrombotic treatment

To determine the best therapeutic approach in individuals with non-valvular AF, the risk of both, stroke and bleeding should be determined in every patient, including the elderly population. All patients aged >75 years with AF have an individual yearly risk of stroke >4 % [6]. However, their risk of hemorrhage is also increased. In fact, age has been included in CHA_2_DS_2_-VASc (1 point for age 65-74 years; 2 points for age ≥75 years) and HAS-BLED (1 point for age >65 years) scores [6, 12, 13]. Despite of this, unless the risk of bleeding is exceedingly high, anticoagulation is required for most elderly subjects [6].

Different studies have shown that anticoagulation is much more effective than antiplatelet therapy to reduce the risk of stroke in patients with AF at risk of arterial thromboembolic complications, particularly in elderly patients. In the ACTIVE-W trial, warfarin was superior to clopidogrel plus aspirin for the prevention of vascular events in patients with AF at high risk of stroke, especially in those subjects already taking oral anticoagulation therapy [14]. In the BAFTA study, in 973 AF patients aged 75 years or over, after a mean follow-up of 2.7 years, treatment with warfarin was associated with a 52 % reduction in the risk of fatal or disabling stroke, intracranial haemorrhage, or clinically significant arterial embolism, compared with aspirin 75 mg daily [15]. In the AVERROES study, in which 5,599 patients with AF (mean age 70 years) who were at increased risk for stroke and for whom VKA therapy was unsuitable, apixaban reduced the risk of stroke or systemic embolism by 55 % compared to aspirin (81 to 324 mg per day), without a significant increase in the risk of major bleeding [16]. Importantly, it has been reported that acetylsalicylic acid has no discernable protective effect against stroke and may even increase the risk of ischaemic stroke in elderly patients [17].

In summary, except when there is a high risk of bleeding, anticoagulation is required in most elderly patients with AF to prevent the risk of stroke. If anticoagulation is contraindicated due to the very high risk of bleeding, the use of antiplatelet drugs instead of anticoagulants is not justified. Antiplatelet agents should only be considered in those elderly patients with AF who reject taking anticoagulants and have concomitant vascular disease. Finally, as these patients have an elevated risk of bleeding, it is essential to identify those factors that increase the risk of hemorrhage (i.e. high blood pressure, concomitant use of non-steroidal anti-inflammatory drugs, alcohol abuse, etc.) in order to modify them to reduce this risk.

### Management of antithrombotic treatment in patients with high risk of gastrointestinal bleeding

Due to age, comorbidities, and polymedication, patients with AF have an increased risk of gastrointestinal bleeding, a risk that is increased by antithrombotic treatment. Treatment with VKA is associated with a three-fold increase in risk of gastrointestinal hemorrhage. The concomitant use of antiplatelets and VKA doubles the risk compared with VKA alone [18–21].

Different meta-analyses have shown that, compared with warfarin, new direct oral anticoagulants (DOACs) provide significant reductions in stroke, intracranial haemorrhage, and mortality, with similar major bleeding risk and higher risk of gastrointestinal hemorrhages [22–25], a finding not apparently observed in individuals aged 75 years and older [26]. Of note, not all DOACs have the same risk of gastrointestinal bleeding, being apixaban the DOAC with the lesser risk [25, 27–29]. In case of patients with history or at high risk of gastrointestinal bleeding, vitamin K antagonists should be considered.

To reduce the risk of gastrointestinal bleeding in patients taking anticoagulants, in those patients treated with VKA, INR levels should be kept within the recommended range over time. In case of treatment with DOACs, dosage should be carefully taken according to age (dabigatran and apixaban), creatinine clearance (all DOACs) and previous history of gastrointestinal bleeding. In addition, the use of non-steroidal anti-inflammatory drugs, antiplatelet agents as well as alcohol use should be avoided [20, 30–34].

### Antithrombotic treatment in patients with anemia or thrombocytopenia

It has been reported that decreased kidney function and lower hemoglobin levels are associated with an increased risk for new-onset AF, especially when both are present [35]. On the other hand, thrombocytopenia is associated with an increased risk of hemorrhage [36, 37]. In HAS-BLED, bleeding refers to previous bleeding history and/or predisposition to bleed (i.e. bleeding diathesis, anemia, etc.) [6]. As a result, before beginning anticoagulation, both conditions should be investigated and corrected, if possible.

Anticoagulation is generally contraindicated if platelet count is <50x10^9^/L. In ROCKET-AF, patients with hemoglobin level <10 g/dL or platelet count < 90.000/μL were excluded from the study [27]. In AVERROES and RELY studies, patients with hemoglobin level <10 g/dL or platelet count <100.000/mm^3^ were also excluded [16, 28]. Therefore, in patients with platelet count >100,000/dL, anticoagulation can be normally prescribed. However, if platelet count is between 50,000 and 100,000/dL, the risk/benefit ratio should be carefully individualized.

Anemia predicts thromboembolic events, bleeding complications and mortality in patients with atrial fibrillation [38]. Although anemia by itself should not be considered as an absolute contraindication for initiating anticoagulation, it requires strict control and follow-up. In fact, the history of anemia increases the risk of warfarin interruption or discontinuation [39]. In patients with mild anemia, it may be recommended to perform a hemogram every 8-12 weeks immediately after anticoagulation is started, and then at least every 6-12 months. If hemoglobin levels remain stable, anticoagulation could be continued.

### Anticoagulation and renal function

Chronic kidney disease is common in patients with AF, particularly in those individuals with permanent AF or a CHA_2_DS_2_-VASc ≥2. A recent study showed that approximately one third of patients with CHA_2_DS_2_-VASc ≥2 had moderate renal dysfunction [40]. Renal insufficiency increases both, the risk of thromboembolic and bleeding outcomes in patients with AF [41]. However, although renal insufficiency is included in bleeding risk scores (i.e. HAS-BLED), it has not been included in stroke risk scores (i.e. CHA_2_DS_2_-VASc) [42, 43]. Remarkably, renal dysfunction does not contraindicate anticoagulation [44].

Dabigatran is contraindicated in patients with creatinine clearance <30 ml/min and rivaroxaban, apixaban and edoxaban are not recommended in subjects with creatinine clearance <15 ml/min. DOACs can be safely used in patients with moderate renal dysfunction, but dose adjustment is required. In individuals with mild impairment or normal renal function, no dose adjustment is needed [45–47]. VKA can be used regardless of renal impairment, and dose is adjusted only according to INR values [48].

Renal function should be determined before starting treatment with DOACs, and then at least every year, and more frequently (at least once every 6 months) when renal dysfunction exists, worsening renal function is suspected, or in case of compromised patients, such as the elderly or frailty [6, 49, 50].

### Cognitive status and antithrombotic therapy

The risk for AF and cognitive disorders increases with age. Different studies have reported a relationship between increased risk for cognitive impairment, dementia and cardiovascular diseases, likely due to embolic strokes or chronic cerebral hypoperfusion [51, 52]. AF increases the risk of dementia. It has been estimated that patients with AF have a 1.7 to 3.3 greater risk of cognitive impairment, and a 2.3-fold increased risk of dementia, compared to patients in sinus rhythm [53]. Different mechanisms may explain this relationship. The concomitant presence of other cardiovascular risk factors (i.e. hypertension, diabetes, obesity, hypercholesterolemia or smoking) increases the risk of new-onset AF and stroke, and both, the risk of dementia. In addition, AF is associated with silent cerebral infarctions and reduced brain volume [54–58].

A number of studies have shown that many patients with cognitive impairment and AF are not anticoagulated. However, dementia by itself should not be considered as an absolute contraindication for anticoagulation. Factors such as the severity of dementia, quality of life, life expectancy, and the presence of other comorbidities should also be taken into account. Importantly, these factors as well as the indication of anticoagulation should be periodically reevaluated [59–61].

On the other hand, worsening of cognitive function in patients already taking VKA may be related with a poor INR control. In these patients, DOACs could be of choice. In addition, simplification of treatment with DOACs may be an advantage in patients with cognitive impairment. Finally, the control of cardiovascular risk factors should also be a target in this population [62–66].

### Antithrombotic treatment in patients at risk of falls

Fall history, dependency in daily activities, age ≥75 years and living alone are independent fall predictors in patients with AF [67]. Although frequent falls have been associated with an increased risk of intracranial bleeding and mortality in anticoagulated patients, the overall risk remains low. In fact, this risk is generally lower than the risk of stroke in elderly patients with AF. As a result, risk stratification of stroke and bleeding is mandatory in this population. The history of frequent falls requires the identification of those factors that can be modified to reduce the risk of intracranial hemorrhage (i.e. drugs, orthostatic hypotension, visual impairments, etc.). If these factors are corrected, anticoagulation can be normally prescribed. If not, the decision should be individualized [68–70].

We consider that in patients at risk of frequent falls with a CHADS_2_ score ≥3, the beneficial effect of anticoagulation is higher than the risk of intracranial hemorrhage. However, in patients with a CHADS_2_ score ≥3 that had a post-traumatic intracranial hemorrhage while taking anticoagulants, anticoagulation should be permanently withdrawn. By contrast, in those patients with a CHADS_2_ score <2 and frequent falls, anticoagulation should be avoided.

Compared with warfarin, DOACs have a lesser risk of intracranial hemorrhage (33-69 % risk reduction depending on the drug), including traumatic intracranial bleeding. As a result, it is reasonable to recommend, the use of DOACs over VKA in patients with a high risk of falls [71, 72].

### Importance of blood pressure control

Since uncontrolled hypertension markedly increases the risk of stroke, including hemorrhagic stroke, reducing blood pressure to recommended targets is mandatory in elderly patients with AF [73–76].

Since alert reaction at clinic may be common in elderly patients taking anticoagulants, the use of ambulatory blood pressure monitoring or 24-hour blood pressure monitoring is recommended in this context. Blood pressure should be controlled at office or with ambulatory blood pressure monitoring, at least every month. On the other hand, assuring an adequate treatment adherence is mandatory [73–76].

In patients with blood pressure ≥180 /100 mmHg, the beginning of anticoagulation should be postponed to achieve blood pressure <160/90 mmHg. This reduction should be attained as soon as possible. In patients already taking anticoagulants, this reduction should be achieved within a few days. The final target in elderly patients taking anticoagulants should be always <160/90 mmHg, and preferably <140/90 mmHg when tolerated [77, 78]. Orthostatic hypotension is common in elderly. Antihypertensive treatment should be carefully prescribed in this population in order to avoid orthostatic hypotension [78].

### Impact of polymedication and medication adherence

Polymedication is common in patients with AF, particularly in elderly population. It has been estimated that AF patients take nearly 7 drugs every day [2, 79].

Polymedication and complexity of treatment are associated with poorer medication adherence. It has been demonstrated that non-valvular AF patients treated with once daily dosing regimens have approximately a 26 % higher likelihood of adherence compared with subjects on twice-daily regimens [80].

Interactions with other drugs are common in patients taking VKA, but minimal with DOACs. In fact, up to 40 % of patients taking VKA withdraw treatment after 5 years of therapy. Compared with warfarin, medication adherence is higher in those patients taking DOACs [81–84].

### When should anticoagulation be interrupted?

In patients taking anticoagulants, stroke and bleeding risk stratification should be periodically analyzed, regardless of age. When bleeding risk overcomes the risk of stroke, treatment should be discontinued. This decision should be taken together with patients and family (Table 1). However, it should be noted that the interruption of anticoagulation is associated with an increased risk of death and thromboembolic complications. Finally, anticoagulation in patients with terminal disease is not associated with an improved survival [85–90]. This information should be carefully explained to patients and family.

**Table 1:** Causes of permanent discontinuation and temporary interruption of anticoagulant therapy and conditions that do not justify anticoagulation withdrawal

Temporary interruption	○ Acute major bleeding (life-threatening hemorrhage, bleeding leading to hospital admission or need for blood transfusion).
	○ Before elective surgery.
	○ Before endoscopic procedures with high risk of hemorrhage
Permanent discontinuation	○ Hypersensitivity or intolerance to the drug.
	○ Refusal of patient.
	○ Medication non-adherence.
	○ Poor short-term prognosis.
	○ Advanced or terminal cancer.
	○ Poor functional status with total dependency.
	○ Advanced cognitive impairment.
	○ Lack of social support that assure adequate drug compliance.
	○ High risk of bleeding.
	○ Retinopathy with high risk of bleeding.
	○ Hepatic disease associated with coagulopathy and clinically relevant bleeding risk.
	○ Alcohol abuse.
Conditions that do not justify anticoagulation withdrawal (but caution should be taken).	○ Comorbidities or frailty do not contraindicate anticoagulation. However, life expectancy, functionality or cognitive impairment, among others, should be considered.
	○ Risk of falls.
	○ Age (elderly).
	○ Previous intracranial bleeding is not an absolute contraindication, except when high risk of recurrence persists. If anticoagulation is considered, DOACs should be preferred over VKA.
	○ History of bleeding, particularly when the cause is eliminated.
	○ Need for dual antiplatelet therapy (i.e. after stent implantation).
	○ Concomitant use of nonsteroidal anti-inflammatory drugs.

New direct oral anticoagulants *DOACs*, vitamin K antagonists, *VKA*

Data taken from references #33,85-90

### Utility of cranial computed tomography or magnetic resonance in elderly patients who require anticoagulation

The prevalence of cerebral microhemorrhages, cerebral white matter lesions (leukoaraiosis) and brain infarcts, increases with age and are associated with a greater risk of warfarin-related hemorrhage following ischemic stroke [91–94]. Since DOACs are associated with a lesser risk of intracranial bleeding compared with warfarin [72, 95–98], it is very likely that patients with these alterations would benefit more from DOACs. However, no current data have confirmed this point. As a result, it is not recommended to perform a cranial computed tomography or magnetic resonance in all elderly patients who require anticoagulation. Despite of that, if any of these techniques are performed and these alterations are detected, DOACs would be the treatment of choice.

### Impact of frailty on treatment with DOACs

Up to 20 % of individuals aged ≥ 75 years in Spain are considered as fragile patients [99]. Frailty is more common in elderly, women, widow, and patients with low socioeconomic status, low educational level, comorbidities, polymedication, physical disability or cognitive impairment. Age is the most common reason for not prescribing anticoagulants in frail patients. Compared with non-fragile patients, fragile individuals taking anticoagulants have an increased risk of bleeding, but also a higher risk of stroke and death when not taking them. As a result, frailty by itself should not contraindicate the use of anticoagulants, but particular caution should be taken in this population [99–103].

### Impact of body weight on treatment with DOACs

With regard to body weight, dose adjustment of VKA should be performed according to INR levels, but not to body weight. By contrast, although no dose adjustment is required in patients with body weight <50 Kg taking dabigatran, a strict follow-up is recommended in these patients. In individuals taking apixaban, the recommended dose is 2.5 mg twice daily in patients with at least two of the following characteristics: age ≥ 80 years, body weight ≤ 60 kg, or serum creatinine ≥ 1.5 mg/dL. In patients with body weight <60 Kg or estimated creatinine clearance of 30-50 mL/min, the recommended dose of edoxaban is 30 mg. Dose adjustment of rivaroxaban is not required according to body weight (Table 2) [98, 104–107].

**Table 2:** Dose adjustment of dabigatran, rivaroxaban and apixaban according to age, renal function and body weight

Drug	Age	Renal function	Body weight
Dabigatran	• <75 years: 150 mg b.i.d.	• CrCl ≥50 mL/min: no dose adjustment is necessary.	• No dose adjustment is necessary according to body weight. However, close clinical follow-up is required for patients with a body weight <50 kg.
	• 75-80 years: 150 b.i.d. (110 mg b.i.d. should be considered when the risk of stroke is low and the bleeding risk is high).	• CrCl 30-50 mL/min: the recommended dose is 150 mg b.i.d. (110 mg b.i.d. for patients with high risk of bleeding).	
	• ≥80 years: 110 mg b.i.d.	• CrCl < 30 ml/min: contraindicated.	
Rivaroxaban	• No dose adjustment is required.	• CrCl ≥50 mL/min: 20 mg o.d.	• No dose adjustment is necessary according to body weight.
		• CrCl 15-49 mL/min: 15 mg o.d.	
		• CrCl <15 mL/min: not recommended.	
Apixaban	• Recommended dose: 5 mg b.i.d.	• Recommended dose: 5 mg b.i.d.	• Recommended dose: 5 mg b.i.d.
	• 2.5 mg b.i.d. in case of at least 2 of the following characteristics: age ≥80 years, body weight ≤60 kg, or serum creatinine ≥ 1.5 mg/dL.	• 2.5 mg b.i.d. in case of at least 2 of the following characteristics: age ≥80 years, body weight ≤60 kg, or serum creatinine ≥ 1.5 mg/dL.	• 2.5 mg b.i.d. in case of at least 2 of the following characteristics: age ≥80 years, body weight ≤60 kg, or serum creatinine ≥ 1.5 mg/dL.
	• No dose adjustment is required according to age, unless criteria for dose reduction are met.	• No dose adjustment is necessary in patients with mild or moderate renal impairment, unless criteria for dose reduction are met.	• No dose adjustment is required according to body weight, unless criteria for dose reduction are met
		• CrCl 15-29 mL/min: 2.5 mg b.i.d.	
		• CrCl < 15 ml/min, or dialysis: not recommended.	
Edoxaban	• No dose adjustment is required.	• CrCl ≥50 mL/min: 60 mg o.d.	• Body weight >60 kg: 60 mg o.d.
		• CrCl 15-49 mL/min: 30 mg o.d.	• Body weight ≤60 kg: 30 mg o.d.
		• CrCl <15 mL/min: not recommended.	

*CrCl* creatinine clearance, *b.i.d*. twice daily, *o.d*. once daily

Data taken from references #104-107

### Do DOACs should be preferred over VKA in elderly patients with AF?

Compared with warfarin, DOACs have a favorable risk-benefit profile, with achievement of significant reductions in stroke, intracranial haemorrhage and mortality, but similar major bleeding [25]. Although no specific study has been performed in elderly population, different substudies of pivotal phase-III clinical trials, with more than 30,000 patients aged 75 years or older included, have shown that compared with warfarin, the efficacy and safety of DOACs in elderly patients were consistent with that of the overall population [72, 95–98]. Since DOACs have a wide therapeutic window, a predictable anticoagulant effect and few interactions with other drugs, these drugs may be preferable to VKA in many elderly patients [108, 109].

However, patients included in clinical trials are quite different from real-life patients (polymedication, comorbidities, cognitive impairment, frailty, etc.) [110].

## Conclusions

Elderly patients with AF are at high risk of stroke. The great majority of these patients should receive anticoagulant therapy, unless contraindication. However, AF in the elderly is a complex condition due to the presence of other comorbidities, cognitive disorders, risk of falls and polymedication. This may explain, at least in part, the underuse of anticoagulation reported in this population. DOACs are at least as effective as conventional therapy, but with lesser risk of intracranial hemorrhage. In addition, the simplification of treatment with these drugs may be an advantage in patients with cognitive impairment. As a result, DOACs may be of choice in many elderly patients with AF.

Due to the limited data, there are relevant gaps in the management of elderly patients with AF. This update tried to clarify them (Table 3).

**Table 3:** Main recommendations performed in elderly patients with AF

•	Except when the risk of bleeding is very high, anticoagulation is required to prevent the risk of stroke in elderly patients with AF.
•	Antiplatelet agents should only be considered in those patients who reject taking anticoagulants and have concomitant vascular disease.
•	It is essential to identify those factors that increase the risk of hemorrhage (i.e. high blood pressure, concomitant use of non-steroidal anti-inflammatory drugs, alcohol abuse, etc.) in order to modify them to reduce this risk.
•	To reduce the risk of gastrointestinal bleeding in patients taking anticoagulants, VKA should be carefully controlled over time. In case of treatment with DOACs, dosage should be carefully prescribed according to age (dabigatran and apixaban) weight (dabigatran, rivaroxaban and apixaban) and creatinine clearance (dabigatran, rivaroxaban and apixaban). The use of non-steroidal anti-inflammatory drugs or antiplatelet agents as well as alcohol abuse should be avoided.
•	In patients with platelet count >100,000/dL, anticoagulation can be normally prescribed. If platelet count is between 50,000 and 100,000/dL, risk/benefit ratio should be carefully individualized.
•	Anemia by itself should not be considered as an absolute contraindication for initiating anticoagulation, but a strict control and follow-up should be performed.
•	DOACs can be safely used in patients with moderate renal dysfunction, but dose adjustment is required. VKA can be used regardless renal function.
•	Dementia by itself should not be considered as an absolute contraindication for anticoagulation. Factors such as the severity of dementia, quality of life, life expectancy, and the presence of other comorbidities should also be considered. These factors should be periodically reevaluated.
•	In patients at risk of frequent falls with a CHADS_2_ score ≥3, the beneficial effect of anticoagulation is higher than the risk of intracranial hemorrhage. By contrast, in those patients with a CHADS_2_ score <2 and frequent falls, anticoagulation should be avoided. In this context, it is reasonable to recommend the use of DOACs over VKA.
•	Reducing blood pressure to recommended targets (<160/90 mmHg, preferably <140/90 mmHg when tolerated) is mandatory in elderly patients with AF.
•	It is not recommended to perform a cranial computed tomography or magnetic resonance in all elderly patients who require anticoagulation.
•	Frailty by itself should not contraindicate the use of anticoagulants, but particular caution should be taken in this population

*VKA* vitamin K antagonists, *DOACs* new direct oral anticoagulants
