# Erratum: Antithrombotic treatment in elderly patients with atrial fibrillation: a practical approach

**DOI:** 10.1186/s12872-015-0150-x

**Published:** 2015-11-19

**Authors:** Carmen Suárez Fernández, Francesc Formiga, Miguel Camafort, Jose María Cepeda Rodrigo, Jesús Díez-Manglano, Antonio Pose Reino, Gregorio Tiberio, Jose María Mostaza, on behalf of Grupo de trabajo de Riesgo vascular de la SEMI

**Affiliations:** Hospital Universitario de La Princesa, Grupo de Riesgo Vascular de la SEMI, Madrid, España; Hospital Universitari de Bellvitge, Grupo de Riesgo Vascular de la SEMI, Hospitalet de Llobregat, Barcelona, España; Atrial Fibrillation Unit (UFA), Internal Medicine Department, Hospital Clinic. University of Barcelona. Research Group in Cardiovascular Risk, Nutrition and Aging. Area. ‘August Pi i Sunyer‘Biomedical Research Institute (IDIBAPS), Barcelona, Spain; Hospital Vega Baja de Orihuela, Grupo de Riesgo Vascular de la SEMI, Orihuela, Alicante España; Hospital Royo Villanova, Grupo de Riesgo Vascular de la SEMI, Zaragoza, España; Complexo Hospitalario Universitario de Santiago, Grupo de Riesgo Vascular de la SEMI, Santiago de Compostela, España; Hospital Virgen del Camino, Grupo de Riesgo Vascular de la SEMI, Pamplona, España; Hospital Carlos III, Grupo de Riesgo Vascular de la SEMI, Madrid, España; Servicio de Medicina Interna, Hospital Universitario de La Princesa, C/Diego de León 62, Madrid, 28006 Spain

**Erratum to:***BMC Cardiovascular Disorders* 2015, **15**:143 doi: 10.1186/s12872-015-0137-7

Following publication of the original version [1] of the article for the BMC Cardiovascular Disorders 2015, it was brought to our attention that a few authors’ names were incorrectly published. The author’s names should be presented as ‘Carmen Suárez Fernández’, ‘Jose María Cepeda Rodrigo’ and ‘Antonio Pose Reino’.
